# Theoretical NMR correlations based Structure Discussion

**DOI:** 10.1186/1758-2946-3-27

**Published:** 2011-07-28

**Authors:** Jochen Junker

**Affiliations:** 1 Fundaçã Oswaldo Cruz - CDTS, Rio de Janeiro - RJ, Brazil

## Abstract

The constitutional assignment of natural products by NMR spectroscopy is usually based on 2D NMR experiments like COSY, HSQC, and HMBC. The actual difficulty of the structure elucidation problem depends more on the type of the investigated molecule than on its size. The moment HMBC data is involved in the process or a large number of heteroatoms is present, a possibility of multiple solutions fitting the same data set exists. A structure elucidation software can be used to find such alternative constitutional assignments and help in the discussion in order to find the correct solution. But this is rarely done. This article describes the use of theoretical NMR correlation data in the structure elucidation process with WEBCOCON, not for the initial constitutional assignments, but to define how well a suggested molecule could have been described by NMR correlation data. The results of this analysis can be used to decide on further steps needed to assure the correctness of the structural assignment. As first step the analysis of the deviation of carbon chemical shifts is performed, comparing chemical shifts predicted for each possible solution with the experimental data. The application of this technique to three well known compounds is shown. Using NMR correlation data alone for the description of the constitutions is not always enough, even when including ^13^C chemical shift prediction.

## Findings

Nuclear Magnetic Resonance allied with Elemental analysis or high resolution Mass Spectroscopy are the most common tools used for the structure elucidation of new compounds. The used 2D NMR experiments like COSY, HSQC, and ^13^C-HMBC deliver correlation information between atoms that can be translated into connectivity information. Out of these, correlation information from COSY and HSQC experiments can be transcribed directly into connectivity between atoms. But the ^13^C-HMBC correlations need more attention because of their ambiguity and complexity. Hence the difficulty of the structure elucidation problem depends more on the type of the investigated molecule than on its size [1]. Saturated compounds can usually be assigned unambiguously using mainly COSY and some ^13^C-HMBC data, whereas condensed heterocycles are problematic due to their lack of protons that could show interatomic connectivities. This ambiguity has driven the development of different software packages to aid in the interpretation of the ^13^C-HMBC correlation data [2-20] as much as the development of additional correlation experiments [21,22].

Most of these approaches have in common that they work only based on experimental NMR correlation data. COCON [1,4,23,24] has recently been extended with the capability to create a theoretical NMR correlation data set, based on a molecule's suggested constitution. The theoretical data set is used as input data for the structure elucidation software COCON. The resulting set of constitutional assignments indicates how unambiguous NMR would have been able to describe the originally suggested molecule. The freely accessible online version of COCON (WEBCOCON at http://cocon.nmr.de) offers this analysis as "Alternative Constitutions".

The data derived from the NMR correlation spectra is the result of magnetization transfer via scalar coupling between the atoms in the molecule of interest. Since the scalar coupling is based on the interatomic bonds, the correlation data will reflect those bonds. Hence, a set of all feasible NMR correlation data (theoretical correlation data) can be derived from the molecular constitution. This is done by iteratively looking for all protons in the molecule, then building a list of their atoms in 2-bond and 3-bond distance. From each proton all connectivities are inspected recursively up to three bonds distance. If a carbon is found in a two bond distance, a ^2^*J *and a 1,1-ADEQUATE correlation are added to the list. If a carbon is found in a three bond distance, a HMBC correlation is added to the list, if a proton is found, a COSY correlation is added. In principle ^4^*J *correlations for COSY and HMBC could be generated, as sometimes they are observable in experiments as well. But, COCON can not handle ^4^*J *COSY correlations, therefore those are left out. The generation of ^4^*J *HMBC correlations is not used, because when the HMBC correlations are allowed to be ^4^*J *in the structure generation process, the process takes much more time and many more results are produced. Finally carbon chemical shifts are generated by table lookup, a table reverse generated based on the chemical shift rules that COCON uses. This values are not comparable to a chemical shift prediction, but enough to ensure that COCON will generate the starting structure.

For online use, the MarvinSketch applet from ChemAxon is available for drawing or loading of the molecule. The resulting MDL file contains all atoms, their connectivity and multiplicity information. Based on this file, the recently developed Module "Alternative Constitutions" in WEBCOCON generates atomtypes, theoretical correlation data and table-based carbon chemical shifts.

The actual magnitude of the scalar coupling, and therefore the observability of a correlation, depends on the atoms involved, their chemical environment and relative geometry. For ^1^*J *and ^2^*J *couplings mainly the atoms involved and their chemical environment are of importance, since the geometry varies little. That is different with ^3^*J *coupling, which depends on the dihedral angle, hence the actual molecular conformation decides on the magnitude of the coupling. The creation of theoretical correlation data disregards the molecule's real conformation, assuming that all correlations are observable. Hence the data set represents the upper limit of correlations that may be experimentally available for the constitution.

**Figure 1:**
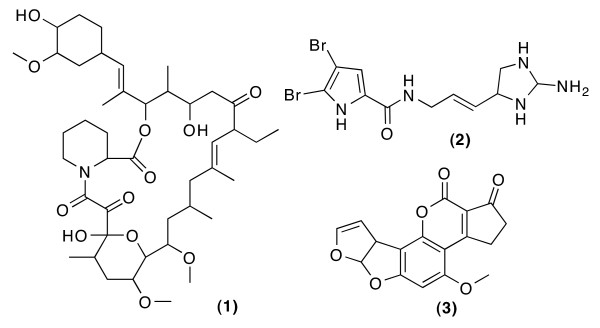
**Ascomycin 1, Oroidin 2 and Aflatoxin B1 3 are used to evaluate the use of theoretical data**.

Calculations were run with three molecules (Figure 1) on the publicly available WEBCOCON server, running times varied from one to twelve minutes. All molecules were drawn in the "Alternative Constitutions" module and submitted to the server. The number of solutions suggested for Ascomycin **1 **and Oroidin **2 **in runs with theoretical and experimental data are shown in table 1. Also, a webpage allowing direct access to the results shown here has been set up on the WEBCOCON server at http://cocon.nmr.de/StructureDiscussion/ (The results are mirrored at http://science.jotjot.net/StructureDiscussion/).

**Table 1:** Number of constitutional assignments suggested for 1 and 2.

	open atom types		fixed atom types	
		
	theo	exp	theo	exp
1	1	100	1	1
2	16	252.566	1	1486

Ascomycin **1 **is a well known ethyl derivative of Tacrolimus, it serves as example of a large natural product, featuring 43 Carbon atoms. Using theoretical NMR correlation data (COSY and ^13^C-HMBC correlations) COCON generates only one solution, independent of whether atom types are defined or not. Using experimental COSY and ^13^C-HMBC correlation data the structure generator comes up with 100 structural assignments, which are reduced to one when the atom types are fixed as well. In this case NMR correlation data was able to define the constitution unambiguously.

Oroidin **2 **has been frequently used for the demonstration of COCON. The use of theoretical COSY and ^13^C-HMBC correlations leads to a total of 16 possible constitutional assignments, also predefining the atom types reduces this set to one constitutional assignment. The experimental data set leads to 252,566 structural assignments generated, which reduce to 1,486 when atom types are predefined as well. Hence the structure can not be safely determined by NMR alone. The original structure determination was carried out by chemical derivatization and total synthesis [25,26].

The pictures change with Aflatoxin B1 **3 **with 17 Carbon atoms. Using theoretical COSY and ^13^C-HMBC data alone, COCON generates 1,048 structures, compared to 1,932 solutions using experimental data. When the atom types are predefined, COCON generates 55 constitutional assignments, compared to 108 with experimental data. The molecule set generated contains constitutions with the element cyclobutadiene, a structural element that is very uncommon in natural products. COCON has several built-in rules that eliminate certain constitutional elements, like cyclobutadiene, cyclopropene and peroxides. By default these rules are not used, but in this special case we observed a substantial difference in the number of results.

**Figure 2:**
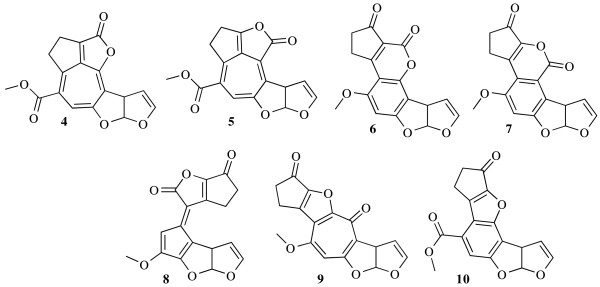
**Planar constitutions suggested for Aflatoxin B1**. Suggestions 4 - 6 are obtained using theoretical data, 5 - 10 using experimental data. Constitution 6 is the correct one.

When these rules are activated the number of solutions drops to 58 for the experimental correlation data set and 33 for the theoretical data set. All planar molecules suggested are shown in Figure 2, the correct constitution and starting point of the analysis is **6**. For the small number of interesting constitutions a back-calculation on the carbon chemical shifts was made (ChemDraw v11), that were compared to the experimental values (see table 2). The last line in the table contains the sum of the absolute chemical shift differences for all carbons, exposing molecule **6 **as the one that best fits the experimental data [24,27,28].

**Table 2:** Experimental and predicted ^13^C chemical shifts for the different constitutions suggested for Aflatoxin B1.

	^13^C shifts for molecule							
	exp	4	5	6	7	8	9	10
C	201	170	167	200	203	202	190	190
C	177	167	166	177	161	167	175	166
C	166	163	163	161	155	154	169	162
C	161	152	154	159	150	145	158	160
C	155	149	149	159	137	144	153	154
C	153	140	142	151	136	141	152	127
C	117	128	125	123	133	126	140	122
C	108	117	116	111	129	122	131	121
C	104	112	113	104	128	107	104	114
CH	145	149	149	149	149	149	149	149
CH	114	106	106	100	105	105	105	107
CH	103	105	105	100	100	94	93	101
CH	91	93	93	97	100	88	93	100
CH	48	43	46	45	48	50	43	45
CH_2_	35	35	35	34	30	32	33	33
CH_2_	29	24	21	28	25	31	14	14
CH_3_	57	53	53	56	56	59	58	52

∑|Δ*δ*|		130	127	**56**	171	122	116	129

The theoretical NMR correlation dataset is the upper limit of number of correlations that are possible with a given constitution. Therefore all alternative constitutions generated with this data are "NMR-identical" with regard to correlation data. A careful analysis of this alternatives might be used to direct further investigations needed to confirm the proposed constitution. Whilst Ascomycin's structure can be confirmed by NMR correlations, Oroidin's structure can not. The results obtained would direct further work towards chemical derivatization and synthesis [25,26] or x-ray crystallography. The results obtained for Aflatoxin B1 show nicely how carbon chemical shift prediction can be used as tool for the structure discussion, exposing one suggested constitutional assignment as best fitting.

## Availability

The WEBCOCON server is freely accessible via http://cocon.nmr.de.

## Competing interests

The author declares that they have no competing interests.

## Authors' contributions

JJ maintains the WEBCOCON software and has run all the calculations shown.
